# Study on the Mechanism of Low-Intensity Pulsed Ultrasound in Ameliorating Glucose Metabolism Through Attenuation of Skeletal Muscle Atrophy in Mice with Type 1 Diabetes

**DOI:** 10.3390/biology14101343

**Published:** 2025-10-01

**Authors:** Zhanke Ma, Yanan Yu, Mengshu Cao, Fang Pang, Lijun Sun, Chenghui Wang, Xiushan Fan, Liang Tang

**Affiliations:** 1 Institute of Sports Biology, Shaanxi Normal University, Xi’an 710119, China; 82001014@nxnu.edu.cn (Z.M.); limonitenan@foxmail.com (Y.Y.); caomengshu@snnu.edu.cn (M.C.); pangfang@snnu.edu.cn (F.P.); sunlijun@snnu.edu.cn (L.S.); 2 Physical Education Institute, Ningxia Normal University, Guyuan 756099, China; 3 Institute of Shaanxi Key Laboratory of Ultrasonics, Shaanxi Normal University, Xi’an 710119, China; wangld001@snnu.edu.cn

**Keywords:** low-intensity pulsed ultrasound (LIPUS), myostatin (MSTN), quadriceps, type 1 diabetes

## Abstract

Diabetic skeletal muscle atrophy is one of the most serious complications among diabetes-related complications. LIPUS enhances muscle regeneration and repair in skeletal muscle injuries. However, whether LIPUS can improve skeletal muscle atrophy in mice with T1DM has not been studied. This study involves forty male C57BL/6 mice randomly divided into four groups: normal control group (NC), streptozocin (STZ)-induced T1DM mice (T1D), T1DM mice treated with LIPUS (DL), and T1DM mice treated with insulin (DI). The DL group was treated on the quadriceps of mice with LIPUS (1 MHz, 80 mW/cm^2^, 20 min/day) for 6 weeks. The results demonstrated that LIPUS significantly improved muscle function by increasing the cross-sectional area, mass, and strength of skeletal muscles. In addition, LIPUS significantly effectively lowered the blood glucose levels of T1DM mice. The knockout of myostatin (MSTN) (MSTN^−/−^) and knockin of MSTN (MSTN^+/+^) mice were employed to verify the underlying mechanism. The results indicated that LIPUS reduces blood glucose levels in T1DM mice by improving their muscle atrophy. This study demonstrated that LIPUS will become a novel therapy for the treatment of skeletal muscle atrophy caused by T1DM.

## Simple Summary

Diabetic skeletal muscle atrophy is a serious complication of diabetes that significantly impairs patients’ quality of life. This study aims to investigate whether low-intensity pulsed ultrasound (LIPUS) can improve skeletal muscle atrophy in mice with type 1 diabetes mellitus (T1DM). Our results showed that LIPUS significantly improved muscle regeneration and repair by increasing skeletal muscle cross-sectional area, mass, and strength. In addition, LIPUS effectively lowered blood glucose levels in T1DM mice. Further experiments indicated that LIPUS reduces blood glucose levels in T1DM mice by improving their muscle atrophy. This study provides new insights into the potential therapeutic application of LIPUS in diabetic skeletal muscle atrophy.

## 1. Introduction

T1DM is characterized by autoimmunity against pancreatic β cells, resulting in their destruction and patients’ subsequent dependency on lifelong insulin replacement [1]. The rising incidences and diagnosis rates have heightened clinical attention to T1DM, emphasizing the urgency of developing targeted interventions. The prevalence of diabetes mellitus increases the burden on the healthcare system and reduces quality of life among patients [2]. The discovery of an effective therapy for this disease remains a major challenge for global public health. Among the various complications of T1DM, skeletal muscle is a major target tissue of diabetic damage [3]. Skeletal muscle, the body’s largest metabolic organ, facilitates movement during exercise and serves as an important reservoir of glucose and protein [4]. At the same time, the skeletal muscle shows a high adaptability to various physiological demands due to the properties of myofibers, which play a key role in metabolism [5]. Additionally, skeletal muscle functions as an endocrine organ that influences the health of other organs and is a critical factor in strength, endurance, and physical performance [6]. However, skeletal muscle atrophy reduces the cross-sectional area of myofibers and weakens muscle strength, increasing the risk of secondary diseases such as osteoporosis and metabolic dysfunction [7]. Therefore, maintaining skeletal muscle health is essential for the body’s fitness.

Exercise training is widely recognized as an effective intervention for maintaining physical fitness. Studies have demonstrated that regular, moderate exercise reduces the risk of obesity, diabetes, cancer, cardiovascular disease, and osteoporosis [8]. Among exercise modalities, resistance training is one of the most effective strategies for slowing the loss of muscle mass and maintaining or increasing strength. However, only about one-third of people with T1DM meet the recommended exercise targets [9]. The main obstacle for T1DM patients is the fear of hypoglycemia [10], which can lead to unpleasant symptoms (e.g., dizziness), serious complications (e.g., loss of consciousness or seizures), and impaired physical and cognitive performance [11,12].

LIPUS is a form of mechanical energy that can be transmitted into biological tissues as a high-frequency acoustic mechanical perturbation [13,14]. Previous studies have shown that the mechanical effects of ultrasound enhance the circulation and distribution of nutrients, oxygen, and signaling molecules within tissues [15]. LIPUS-induced mechanical stimulation mimics exercise-like physiological effects. Meanwhile, it has established therapeutic benefits, including accelerated muscle growth [16] and prevention of bone loss [17].

MSTN, a member of the TGF-β superfamily, is a key negative regulator of skeletal muscle growth; its decline leads to a dramatic increase in muscle mass, a finding corroborated by our group’s prior research [18]. Conversely, elevated MSTN levels in humans are associated with reduced skeletal muscle quality and strength. Studies in mice further reveal that skeletal muscle atrophy correlates with elevated MSTN expression [18]. Consequently, MSTN expression serves as a biomarker of skeletal muscle atrophy and has emerged as a promising therapeutic molecular target for muscle-related disorders.

Previous study demonstrated that skeletal muscle plays a critical role in maintaining glucose homeostasis during insulin-stimulated conditions [18]. Uncontrolled T1DM is associated with significant muscle loss [19], exacerbating hyperglycemia and perpetuating a detrimental metabolic cycle. However, whether LIPUS simultaneously alleviates skeletal muscle atrophy and improves diabetic symptoms in T1DM mice remains unclear. We hypothesize that LIPUS mitigates T1DM symptoms by reducing skeletal muscle atrophy, thereby improving glycemic control.

We investigated the relationship between skeletal muscle atrophy and LIPUS through an STZ-induced T1DM mice model. The skeletal muscle atrophy-alleviating effects of T1DM mice produced by LIPUS were evaluated. In addition, the potential molecular mechanisms were investigated by analyzing the expression of MSTN, Akt, mTOR, GSK-3β, and GLUT4 at both gene and protein levels.

## 2. Materials and Methods

### 2.1. Animals

Eight-week-old male C57BL/6J mice were purchased from the experimental animal breeding research center of Xi’an Jiaotong University (Xi’an, China). Furthermore, C57BL/6J mice (MSTN^−/−^, MSTN^+/+^, Cyagen Biotechnology Co. Ltd., Suzhou, China) were employed to evaluate the relationship between muscle atrophy and LIPUS. To identify the transgenic mice, genomic DNA was first extracted from the tail or tissue and then amplified by PCR with specific primers. The genotype was determined by a combination of agarose gel electrophoresis and sequencing detection. Meanwhile, tissue protein extracts were incubated with specific antibodies and then analyzed for colorimetric changes to observe the variation in MSTN protein expression. By integrating the results from the gene and protein level, it was possible to precisely identify the transgenic mice. Ultimately, MSTN^−/−^ (*n* = 5), MSTN^+/+^ (*n* = 5), and wild-type male mice (*n* = 5) were selected for the subsequent experimental studies. All mice were kept at a temperature of 22–25 °C (60 ± 5% humidity, 12 h light/dark cycle). The body weights of the mice were measured every week during the experiment. All procedures were approved by the Animal Ethics Committee of Shaanxi Normal University (Approval No.202516062) and performed in accordance with the National Institute of Health Laboratory Animal Care and Use Guidelines (NIH Publication No. 8023, revised in 1978). All mice had free access to the required food and water.

### 2.2. Animal Grouping and Treatments

Animals were randomly divided into 4 groups (*n* = 10 per group) after a week of acclimatization: normal control group (NC), STZ-induced T1DM mice (T1D), T1DM mice treated with LIPUS (DL), and T1DM mice treated with insulin (DI). The mice in the diabetes groups received intraperitoneal injections of STZ (Sigma-Aldrich, Cat. No. 18883664, St. Louis, MO, USA) prepared in citrate buffer (0.1 M, pH = 4.5) at a dose of 60 mg/kg/day for 5 days [20].

The NC mice received the same dose of sodium citrate buffer to exclude false-positive results. After STZ administration, the T1DM model was confirmed by measuring fasting blood glucose (FBG) in tail vein blood samples [18]. Meanwhile, the body weight, FBG levels, and food and water intake of the mice were recorded weekly during the experiment. After the T1DM model was established, 6-week insulin injection and LIPUS treatments were performed for the mice. The mice in the DI group were given a subcutaneous insulin injection (0.5–1 U/day) at the same time every day over the course of the next 6 weeks. Before each insulin injection, the dosage of insulin was adjusted to the non-fasting glucose level. A self-developed LIPUS device (designed and manufactured by Intelligent Medical Ultrasound Lab of Fudan University, Shanghai, China) was used for animal therapy. The mice involved in the LIPUS treatment [17] (frequency: 1.5 MHz; duty cycle: 20%; pulse repetition frequency: 1 kHz; intensity: 80 mW/cm^2^, respectively) were exposed to LIPUS on the quadriceps femur for 20 min/day for 6 weeks.

### 2.3. FBG Analysis [18]

FBG was assessed after a 12 h overnight fast. Animals were briefly restrained without anesthesia, and the tail tip was sterilized with 70% ethanol and subsequently pierced with a sterile 21-gauge lancet. The first drop of blood was wiped away to eliminate tissue fluid contamination; the second drop (≈1 µL) was immediately applied to the test strip of a GLM-76 analyzer (Qingdao Houde Biotechnology Co., Ltd., Qingdao, China). Readings were recorded once the instrument signaled stability (within 5 s). FBG was measured every week during the experiment.

### 2.4. Oral Glucose Tolerance Tests (OGTTs) [18]

OGTTs were performed by testing the blood in the tail veins of the mice. After the mice had fasted for 12–14 h, blood samples were collected at 0, 15, 30, 60, 90, and 120 min after oral administration of glucose (2 g/kg) for the OGTT analysis. Blood glucose levels were measured using an Accu Check glucose analyzer (Roche Diabetes Care GmbH, Raunheim, Germany) via tail puncture at predetermined time intervals. The results were expressed as the area under the curve (AUC).

### 2.5. Grip Strength Test [18]

The forelimb strength of mice was assessed weekly by a grip strength meter (YLS13A, Anhui Zhenghua Biological Instrument Equipment Co., Ltd., Huaibei, Anhui, China). Each measurement was performed in triplicate to minimize variability, and the final value was calculated as the mean of three trials.

### 2.6. Quadriceps Femoris Muscle Contraction In Vivo and In Situ Test and Maximum Tensile Load of Intestine

Quadriceps femoris contractility was gauged in vivo with a 1305A apparatus (Aurora Scientific, Aurora, ON, Canada). After fur removal, the supine mouse was secured on a 37 °C platform and the contralateral foot was taped to prevent movement. Paired 0.5 mm steel electrodes were inserted subcutaneously at the proximal and distal ends of the muscle. A 100 Hz, 0.2 ms, 500 ms tetanic train evoked a fused contraction; three trials (3 min apart) were averaged for peak force. The in situ test method for quadriceps femoris muscle contraction followed a similar procedure. Surgical sutures were used to secure the distal end of the quadriceps femoris muscle, and a lamp was utilized to maintain the experimental temperature at 37 °C. Paired 0.5 mm steel electrodes were inserted subcutaneously at the proximal and distal ends of the muscle. A 100 Hz, 0.2 ms, 500 ms tetanic train evoked a fused contraction; three trials (3 min apart) were averaged for peak force.

### 2.7. Morphometric Analysis [17,18]

Tissue processing was performed rapidly to preserve tissue integrity. Following weighing, quadriceps tissue was fixed immediately in 4% paraformaldehyde to preserve structural integrity. Fixed tissues were embedded in paraffin and sectioned into 4 μm thick slices. Sections were stained with hematoxylin and eosin (H&E) to facilitate histological examination under a microscope. Image analysis software (ImageJ 1.54p) was utilized to measure the average cross-sectional area of muscle fibers at 200× magnification. Finally, images were acquired using an inverted optical microscope (Olympus BX-51, Tokyo, Japan) and subjected to histomorphometric analysis. Quadriceps femoris muscle (<1 mm^3^) was fixed 2 h in 2.5% glutaraldehyde (0.1 M cacodylate, pH 7.4), post-fixed 1 h in 1% OsO_4_, dehydrated, and embedded in Spurr’s resin. Ultrathin sections (70 nm) were uranyl/lead stained and imaged at 200 kV (Talos F200C, Thermo Fisher Scientific, Brno, Czech).

### 2.8. Serum Analysis

Biochemical reagent kits, sourced from Nanjing Jiancheng College of Biotechnology (Nanjing, China), for succinate dehydrogenase (SDH), malate dehydrogenase (MDH), and glycated serum protein (GSP) were utilized to determine their respective activities in mouse serum. The testing procedure, including all specific steps, was meticulously followed in accordance with the manufacturer’s instructions provided with each reagent kit. Absorbance readings were obtained using a Model 680 microplate reader manufactured by Bio-Rad Corp (Philadelphia, PA, USA). Insulin and MSTN levels were quantified via ELISA (EMD Millipore, Burlington, MA, USA).

### 2.9. Western Blot Analysis

Quadriceps femoris muscle tissue samples (50 mg) were accurately weighed and homogenized in 500 μL RIPA lysis buffer. Protein concentration was determined using a BCA protein assay kit (Thermo Fisher Scientific, Waltham, MA, USA). Total protein was resolved on 8–12% SDS-polyacrylamide gels (SDS-PAGE) and transferred onto polyvinylidene fluoride (PVDF) membranes. For immunoblotting, membranes were incubated overnight at 4 °C with primary antibodies (Cell Signaling Technology, Beverly, MA, USA), followed by 1 h incubation with horseradish peroxidase (HRP)-conjugated secondary antibodies at room temperature. Protein signals were detected using an enhanced chemiluminescence (ECL) system (Amersham, Little Chalfont, UK). Protein bands were imaged using an Azure Biosystems C300 imaging system (Azure Biosystems, Dublin, CA, USA) and quantified via densitometry using Bio-Rad Quantity One software (Bio-Rad, Hercules, CA, USA). Glyceraldehyde-3-phosphate dehydrogenase (GAPDH) served as the loading control. The primary antibodies included the following: myostatin (MSTN; EPR4567(2), ab124721) from Abcam (Cambridge, UK); GLUT4 (2213S), AKT (9272S), and mTOR (2972S) from Cell Signaling Technology (Danvers, MA, USA); and GSK-3β (bsm-33294M) from Bioss Biotechnology Co., Ltd (Beijing, China).

### 2.10. Statistical Analysis

The datasets were analyzed for normality and homogeneity of variance, and parametric post hoc statistical analysis was performed to confirm a priori power calculations. Statistical analysis was performed using SPSS version 26.0 (IBM, Armonk, NY, USA) and Graph Pad Prism 8.0 software (Graph Pad, Inc., La Jolla, CA, USA). Univariate analysis of variance between the four groups was performed and the significance between each group was determined using Tukey’s multiple comparison test. *p* < 0.05 was considered statistically significant.

## 3. Results

### 3.1. Physiological Characteristics of Mice

**Figure 1:**
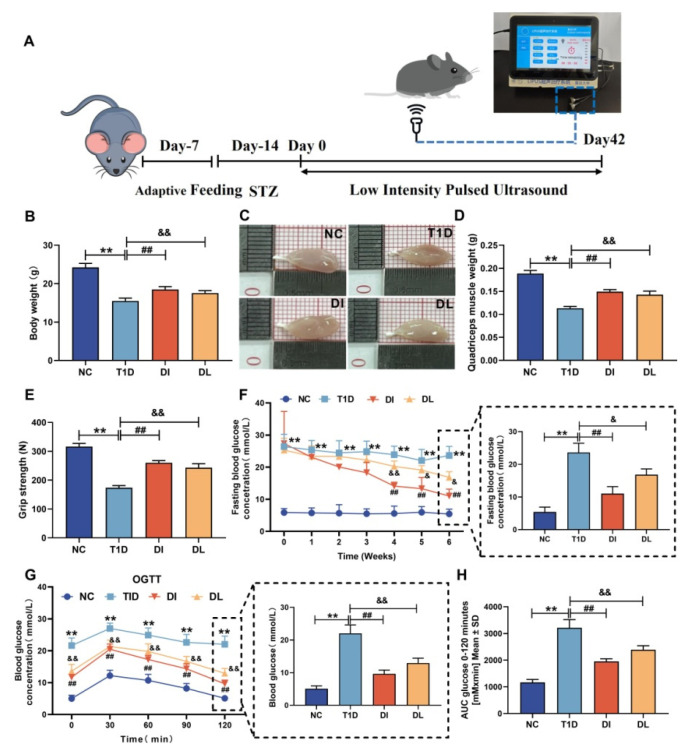
Physiological and serum characteristics of mice. Schematic diagram of experimental protocol (**A**), body weight (**B**), morphology of quadriceps (**C**), quadriceps muscle weight (**D**), grip strength (**E**), FBG (**F**), OGTT (**G**), and AUC of blood glucose (**H**). The data are expressed as mean ± SD (NC vs. T1D: ** *p* < 0.01; T1D vs. DI: ## *p* < 0.01; T1D vs. DI: & *p* < 0.05 and && *p* < 0.01).

The T1DM mouse model was established by a single intraperitoneal injection of STZ, and the experimental protocol is shown in Figure 1A. After 2 weeks of STZ administration, mice in the T1D group showed a significant reduction in body weight, quadriceps femoris weight, and grip strength compared with NC group. Compared with the T1D group, the DI and DL groups showed a significant increase in body weight, muscle weight, and grip strength (both *p* < 0.01, Figure 1B–E). Figure 1F shows the FBG results. Compared with the NC group, the T1D group showed a significant increase in FBG (*p* < 0.01). However, compared with the T1D group, the DI and DL groups showed a significant decrease in FBG (*p* < 0.01 and *p* < 0.05). Figure 1G shows OGTT. Compared with the NC group, the T1D group showed a significant increase in OGTT (both *p* < 0.01). However, compared with the T1D group, the DI and DL groups showed a significant decrease in OGTT (both *p* < 0.01). Figure 1H shows AUC of blood glucose. Compared with the NC group, the T1D group showed a significant increase in AUC of blood glucose (*p* < 0.01). However, compared with the T1D group, the DI and DL groups showed a significant decrease in the AUC of the blood glucose (*p* < 0.01 and *p* < 0.01).

### 3.2. Serum Analysis

**Figure 2:**
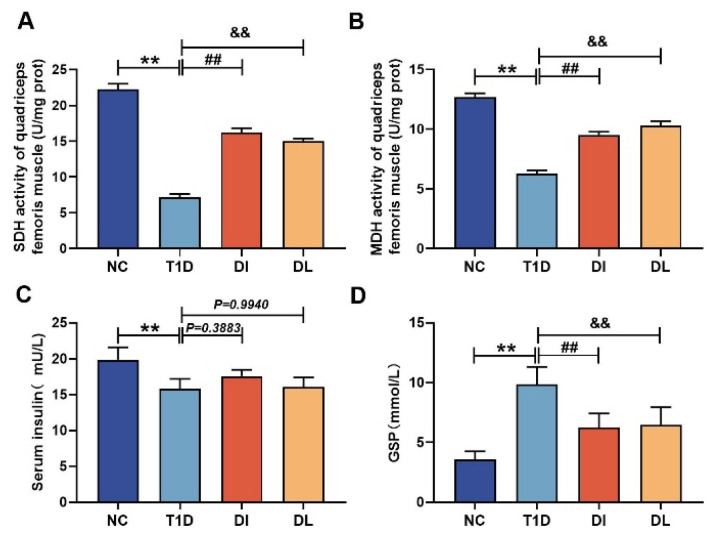
The effects of LIPUS on the quadriceps femoris of diabetic mice SDH (**A**), MDH (**B**), insulin (**C**), and GSP (**D**) activity. The data are expressed as mean ± SD (*n* = 3. NC vs. T1D: ** *p* < 0.01; T1D vs. DI: ## *p* < 0.01; T1D vs. DI: && *p* < 0.01).

The effects of LIPUS on the activity levels of the key muscle metabolism enzymes, including SDH and MDH, were investigated. Compared with the NC group, the activity levels of SDH and MDH in the T1D group were significantly decreased. However, compared with the T1D group, the DI and DL groups showed a significant increase in SDH and MDH levels (both *p* < 0.01, Figure 2A,B). Figure 2C shows insulin levels. The T1D group exhibited a significant decrease in insulin levels compared to the NC group (*p* < 0.01). Importantly, no significant changes were observed in the DI and DL groups (*p* = 0.3883 and *p* = 0.9940). Figure 2D shows glycated serum protein (GSP) levels. The T1D group exhibited a significant increase in GSP levels compared to the NC group (*p* < 0.01). However, compared with the T1D group, the DI and DL groups showed a significant decrease in GSP levels (both *p* < 0.01).

### 3.3. Morphological Analysis

**Figure 3:**
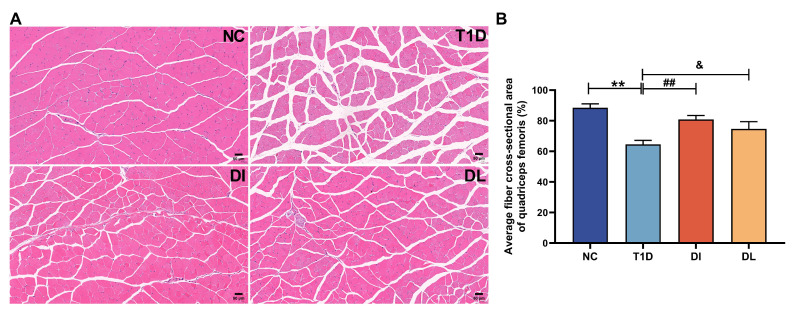
Effect of LIPUS on quadriceps femoris cross-sectional area in diabetic mice H&E staining of quadriceps femoris (**A**); average fiber cross-sectional area of quadriceps femoris (**B**). The data are expressed as mean ± SD. Each sample represents six different fields of view; images were taken at ×200 under a light microscope. (*n* = 3. NC vs. T1D: ** *p* < 0.01; T1D vs. DI: ## *p* < 0.01; T1D vs. DI: & *p* < 0.05).

The results of the HE staining analysis demonstrated significant alterations in muscle fiber morphology. Specifically, the T1D group exhibited irregular muscle fiber arrangement, with widened gaps between adjacent epimysia. In contrast, the NC, DI, and DL groups exhibited orderly muscle fiber arrangement and narrow epimysial spaces (Figure 3A). Compared with the NC group, the average fiber cross-sectional area of the quadriceps femoris in the T1D group were significantly decreased (*p* < 0.01). However, compared with the T1D group, the DI and DL groups showed a significant increase in the average fiber cross-sectional area of the quadriceps femoris (*p* < 0.01 and *p* < 0.05, Figure 3B).

### 3.4. Electron Microscope Analysis

**Figure 4:**
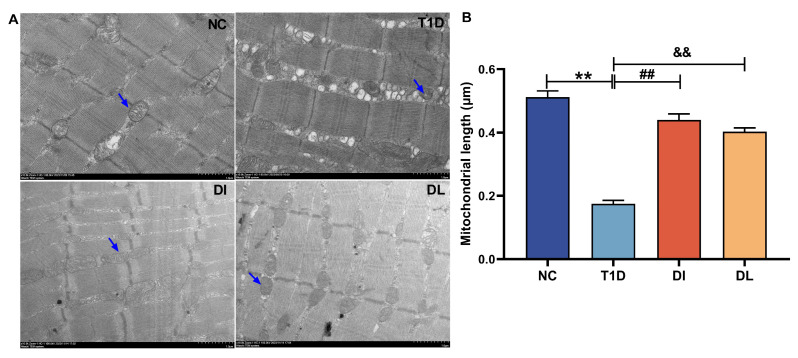
Electron microscope analysis of quadriceps muscle. Electron microscope of muscle, blue arrow: mitochondria (**A**), the length of mitochondrial (**B**). The data are expressed as mean ± SD (*n* = 3. NC vs. T1D: ** *p* < 0.01; T1D vs. DI: ## *p* < 0.01; T1D vs. DI: && *p* < 0.01).

As demonstrated in Figure 4A, transmission electron microscopy reveals significant morphological variations of the muscle. In the NC group, muscle fibers were neatly arranged, mitochondria exhibited regular, oval morphology with intact membranes, and Z-lines were clearly visible. However, the T1D group displayed disorganized muscle fibers, significantly widened interstitial spaces, and mitochondria that were both abnormally increased in number and heterogeneous in size. Mitochondrial fusion resulted in vacuole-like structures, while Z-lines appeared blurred or absent. After LIPUS treatment, muscle fiber gaps were reduced, mitochondrial numbers decreased, and fusion tendencies were observed. Figure 4B shows mitochondrial length, compared with the NC group, mitochondrial length in the T1D group was significantly decreased (*p* < 0.01). However, compared with the T1D group, the DI and DL groups showed a significant increase in mitochondrial length (*p* < 0.01 and *p* < 0.01).

### 3.5. Western Blot Analysis

**Figure 5:**
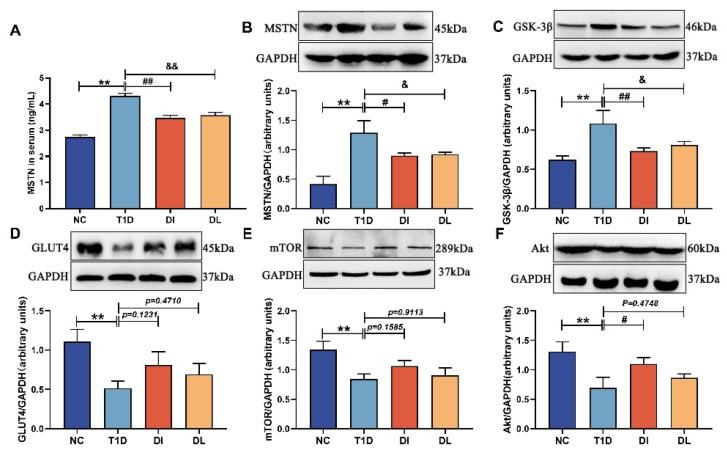
The protein expression of the quadriceps in mice. The protein expression level of MSTN in serum (**A**); the protein expression level of MSTN (**B**), GSK-3β (**C**), GLUT4 (**D**), mTOR (**E**), and Akt (**F**). The data are expressed as mean ± SD (*n* = 3. NC vs. T1D: ** *p* < 0.01; T1D vs. DI: # *p* < 0.05 and ## *p* < 0.01; T1D vs. DI: & *p* < 0.05 and && *p* < 0.01).

Figure 5A shows the serum MSTN activity (The original western blot images can be found in Supplementary Figure S1). The T1D group showed a significant increase in MSTN activities compared to the NC group (*p* < 0.01). The DI and DL groups showed a significant decrease in MSTN activities compared to the T1D group (*p* < 0.01 and *p* < 0.01). As illustrated in Figure 5B,C, the protein expression levels of MSTN and GSK-3β were significantly upregulated in the T1D group compared to the NC group (*p* < 0.01 and *p* < 0.01). The protein expression levels of MSTN and GSK-3β were significantly downregulated in the DI and DL groups compared to the T1D group (*p* < 0.05, *p* < 0.01; *p* < 0.05, *p* < 0.05). As illustrated in Figure 5D–F, the protein expression levels of GLUT4, mTOR, and Akt were significantly downregulated in the T1D group compared to the NC group (both *p* < 0.01). The protein expression levels of GLUT4, mTOR, and Akt were upregulated in the DI and DL groups compared to the T1D group (*p* = 0.1231, *p* = 0.1585, *p* < 0.05; *p* = 0.4710, *p* = 0.9113, *p* = 0.4748).

### 3.6. Transcriptome Sequencing Data Evaluation

This study rigorously assessed the quality of mouse quadriceps sequencing data related to mRNA. We evaluated key indicators of both raw data and clean data (Table 1). Multiple parameters were analyzed, including the number of reads, the total number of bases, and the average sequence length. We also analyzed the quality scores at different thresholds (Q10, Q20, and Q30) and the GC content. The results of high-throughput sequencing demonstrate excellent data quality. Specifically, the percentage of bases with a quality score of Q20 or higher exceeds 99%, the percentage of bases with a quality score of Q30 or higher exceeds 96%, and the GC content remains stably within the suitable range of 48.30% to 19.87%. Further analysis of the distribution of the lengths of clean sequences indicates that the average sequence length falls within the range of 147.36 to 147.49 nucleotides. This narrow range suggests a certain consistency in the sequencing process. These results clearly demonstrate that the quality of the sequencing data meets stringent standards, laying a robust foundation for the subsequent in-depth analysis of the mouse quadriceps data related to mRNA.

### 3.7. The mRNA Expression Analysis

**Figure 6:**
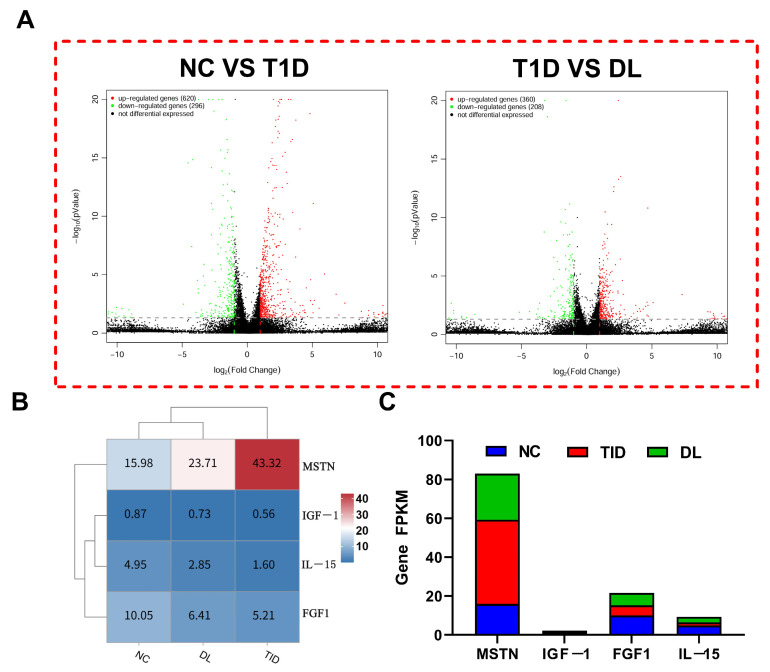
The mRAN expression difference of gene. Volcanic map (**A**); heat map (**B**); Gene FPKM (**C**). Note: red dots indicate the upregulation of genes; green dots indicate the downregulation of genes; black dots indicate the non-differential genes.

This study analyzed gene expression differences and identified the differentially expressed genes (DEGs) associated with diabetic muscle atrophy following LIPUS treatment. As shown in Figure 6A,B, compared with the NC group, the T1D group exhibited significant upregulation of 620 DEGs and downregulation of 296 DEGs. In the LIPUS-treated DL group, 90 DEGs showed significant upregulation and 129 DEGs were downregulated. Compared with the T1D group, the DL group demonstrated significant upregulation of 360 DEGs and downregulation of 208 DEGs. In addition, we analyzed the myokines MSTN, IGF-1, FGF1, and IL-15; among them, MSTN showed the most pronounced change in expression.

### 3.8. Physicochemical Characteristics of MSTN^−/−^ and MSTN^+/+^ Mice

**Figure 7:**
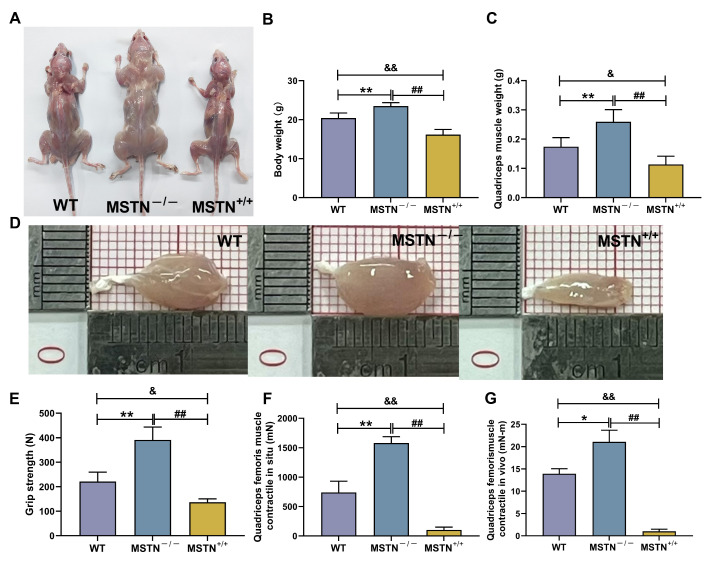
The weight, grip, muscular contraction force, and tissue weight of the MSTN^−/−^ and MSTN^+/+^ mice. The overall appearance (**A**), the weight (**B**), quadriceps femoris morphology (**C**), muscle morphology (**D**), grip strength (**E**), quadriceps femoris muscle contractile in situ (**F**), and quadriceps femoris muscle contractile in vivo (**G**). The data are expressed as mean ± SD (*n* = 5. WT vs. MSTN^−/−^: * *p* < 0.05 and ** *p* < 0.01; MSTN^−/−^ vs. MSTN^+/+^: ## *p* < 0.01; WT vs. MSTN^+/+^: & *p* < 0.05 and && *p* < 0.01).

The physicochemical characteristics of MSTN^−/−^, MSTN^+/+^, and wild-type (WT) mice are demonstrated in Figure 7A. As shown in Figure 7B–D, compared to the WT group, MSTN^−/−^mice exhibited significant increases in body weight and muscle weight (*p* < 0.01, *p* < 0.01). Compared to the MSTN^−/−^group, MSTN^+/+^ mice exhibited significant decreases in body weight and muscle weight (*p* < 0.01 and *p* < 0.01). Compared to the WT group, MSTN^+/+^ mice exhibited significant decreases in body weight and muscle weight (*p* < 0.01 and *p* < 0.05). Figure 7E–G presents functional performance metrics for the quadriceps femoris. Compared to the WT group, MSTN^−/−^ mice exhibited significant increases in skeletal muscle function (*p* < 0.01, *p* < 0.01 and *p* < 0.05). Compared to the MSTN^−/−^ group, MSTN^+/+^ mice exhibited significant decreases in skeletal muscle function (*p* < 0.01, *p* < 0.01, *p* < 0.01). Compared to the WT group, MSTN^+/+^ mice exhibited significant decreases in skeletal muscle function (*p* < 0.05, *p* < 0.01 and *p* < 0.01).

### 3.9. Protein Expression of MSTN^−/−^ and MSTN^+/+^ Mice

**Figure 8:**
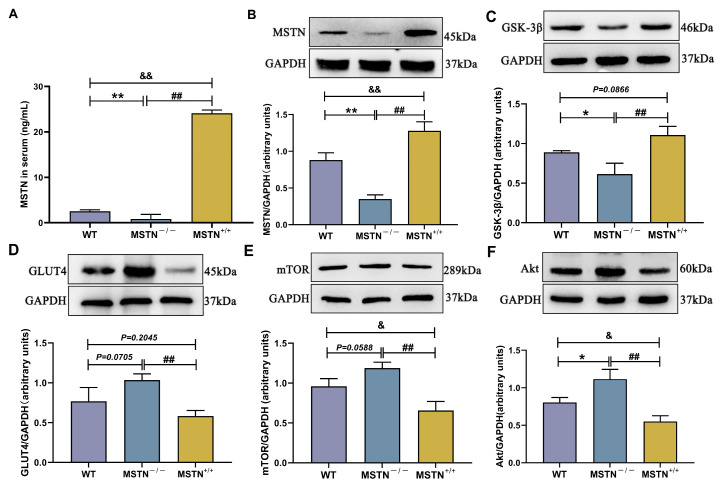
The protein expression of the transgenic mice. The protein expression level of MSTN in serum (**A**); the protein expression level of MSTN (**B**), GSK-3β (**C**), GLUT4 (**D**), mTOR (**E**), and Akt (**F**). The data are expressed as mean ± SD (*n* = 3. WT vs. MSTN^−/−^: * *p* < 0.05 and ** *p* < 0.01; MSTN^−/−^ vs. MSTN^+/+^: ## *p* < 0.01; WT vs. MSTN^+/+^: & *p* < 0.05 and && *p* < 0.01).

Figure 8A shows serum MSTN activity (The original western blot images can be found in Supplementary Figure S2). The MSTN^−/−^ group exhibited a significant decrease in MSTN activity compared with the WT group (*p* < 0.01). The MSTN^+/+^ group showed a significant decrease in MSTN activities compared to the MSTN^−/−^ group (*p* < 0.01). Hower, the MSTN^+/+^ group showed a significant increase in MSTN activities compared to the WT group (*p* < 0.01). As illustrated in Figure 8B,C, the protein expression levels of MSTN and GSK-3β were significantly downregulated in the MSTN^−/−^ group compared to the WT group (*p* < 0.01 and *p* < 0.05). The protein expression levels of MSTN and GSK-3β were significantly upregulated in the MSTN^+/+^ group compared to the MSTN^−/−^ group (both *p* < 0.01). The protein expression levels of MSTN and GSK-3β were upregulated in the MSTN^+/+^ group compared to the WT group (*p* < 0.01 and *p* = 0.0866). As illustrated in Figure 8D–F, the protein expression levels of GLUT4, mTOR, and Akt were upregulated in the MSTN^−/−^ group compared to the WT group (*p* = 0.0705, *p* = 0.0588, and *p* < 0.05). The protein expression levels of MSTN and GSK-3β were significantly downregulated in the MSTN^+/+^ group compared to the MSTN^−/−^ group (both *p* < 0.01). The protein expression levels of MSTN and GSK-3β were downregulated in the MSTN^+/+^ group compared to the WT group (*p* = 0.2045, *p* < 0.05, and *p* < 0.05).

## 4. Discussion

T1DM is an autoimmune disease characterized by immune-mediated targeting of pancreatic β-cells via specific antigens, resulting in β-cell destruction and insulin deficiency. Insulin deficiency impairs glucose uptake in skeletal muscles, contributing to hyperglycemia. Chronic hyperglycemia drives muscle atrophy, impairing muscle function and systemic health [21]. Moreover, T1DM can cause skeletal muscle atrophy. Therefore, inadequate T1DM management imposes significant physical and psychological burdens on patients, reduces quality of life for caregivers, and escalates societal healthcare costs. Previous studies showed that LIPUS promotes muscle cell proliferation and differentiation, facilitates tissue repair, and offers novel therapeutic strategies to improve T1DM [22,23]. Therefore, the effects of LIPUS on STZ-induced diabetic mice explores the mechanisms underlying its enhancement of glucose utilization were investigated in this study.

Following STZ injection, mice exhibited classic diabetic symptoms, including polyuria, polydipsia, polyphagia, and weight loss. Blood glucose levels rose sharply, validating successful model establishment. T1DM necessitates lifelong insulin replacement therapy; discontinuation promotes protein catabolism and complications such as muscle wasting [24,25]. Mice with a FBG concentration persistently ≥16.7 mmol L-1 were considered to have valid T1DM models [18]. After STZ administration, FBG levels remained consistently elevated (25 mmol L^−1^), indicating successful and sustained destruction of pancreatic cells. All STZ-injected mice subsequently developed to T1DM mice by the classic symptoms, including body weight loss, polyuria, and polydipsia. After 6 weeks of LIPUS therapy, the mice exhibited improvements in weight, grip strength, muscle mass, and quadriceps fiber morphology, along with suppressed hyperglycemia, indicating that LIPUS mitigates STZ-induced muscle atrophy.

MSTN, a key negative regulator of muscle growth, contributes to insulin resistance-associated skeletal muscle atrophy. MSTN is synthesized in skeletal muscle, circulates systemically, and promotes muscle degradation. Previous studies [26,27] demonstrate that skeletal muscle MSTN expression is markedly elevated in STZ-induced T1D mouse models, correlating with atrophy progression. LIPUS reduces MSTN protein and mRNA levels in diabetic skeletal muscle, inhibiting the loss of skeletal muscle [28]. Local MSTN propeptide expression enhances glucose transporter activity, improving skeletal muscle glucose utilization and offering novel therapeutic strategies [29]. Our findings confirm elevated MSTN expression in STZ-T1D mice, with LIPUS exerting systemic metabolic benefits via local MSTN inhibition, suggesting MSTN suppression plays a pivotal role in LIPUS-mediated improvements.

The Akt/mTOR signaling pathway regulates critical cellular processes, including growth, adhesion, migration, and survival [30,31,32]. Akt activation influences skeletal muscle hypertrophy and atrophy by promoting proliferation through cell cycle regulators. Akt and its anabolic target, mTOR, drive protein synthesis [33,34]. According to our results for the MSTN/Akt signaling pathways, the protein expressions of MSTN in muscles were decreased by LIPUS in diabetes rats, indicating that the inhibition of MSTN may contribute to improvements in T1DM-induced skeletal muscle atrophy. Previous research has shown that MSTN is a secreted signaling molecule that not only acts to limit muscle mass but also circulates in the blood, where it acts as an endocrine factor [35]. Moreover, local MSTN inhibition by overexpression of its propeptide increases glucose transporter expression and enhances skeletal muscle glucose disposal [29].

By LIPUS treatment, Akt is activated, and initiates two major pathways, including Akt-mediated translocation of GLUT4, and inhibition of GSK-3β. GSK-3β is a key protein kinase in glycogen metabolism that phosphorylates and inhibits glycogen synthase, a key enzyme in glycogen synthesis, and thereby contributes to decrease glycogen storage in the muscle [36]. Elevated levels of GSK-3β in skeletal muscle result in an imbalance in glycogen metabolism, and impaired glucose tolerance. Meanwhile, GLUT4 plays a vital role in regulation of insulin-responsive glucose transport in skeletal muscle, the increase of which correlates with enhanced glucose transport and increased glycogen content [37]. GLUT4 deficiency skeletal muscle is implicated in impaired insulin-stimulated glycogen synthesis in patients with diabetes. Our findings demonstrated that activated AKT activity, as well as decreased protein levels of GSK-3β and increased protein levels of GLUT4 with LIPUS treatment, might have contributed to promoting anabolic pathways and glycogen storage in skeletal muscle.

To further validate our hypothesis, that improving muscle condition in T1DM mice would alleviate blood glucose levels, an MSTN knockout and knockin model for T1DM mice was established. The MSTN^−/−^ mice showed in a rapid increase in muscle mass. Therefore, the increased protein expression of Akt, GLUT4, and mTOR and the decreased protein expression of GSK-3β indicate the amelioration of glucose metabolism and the alleviation of skeletal muscle atrophy capabilities. Meanwhile, the MSTN^+/+^ mice exhibited protein expression changes that are opposite to those in MSTN^−/−^ mice. Collectively, all these demonstrated that LIPUS alleviated glucose levels in T1DM mice through the improvement of skeletal muscle atrophy. LIPUS holds therapeutic potential for simultaneously addressing skeletal muscle atrophy and hyperglycemia, though its biological mechanisms and optimal parameters require further investigation.

## 5. Conclusions

In this study, 6-week LIPUS treatment reduces blood glucose levels by improving the muscle atrophy of T1DM mice. These beneficial effects possibly mediated through the MSTN/Akt/mTOR, MSTN/Akt/GLUT-4, and MSTN/AKT/GSK-3β signaling pathways in skeletal muscle. This study reveals that LIPUS represents a promising non-pharmacological strategy that could serve as an adjunctive or alternative therapeutic approach for the management of T1DM.

## Figures and Tables

**Table 1:** QC data of each group of mouse quadriceps muscle samples.

Sample	Reads Count	Bases Count	N Bases Count	Average Length	Q10 Ratio	Q20 Ratio	Q30 Ratio	GC Ratio
NC	43,210,200	6,367,262,475	351,293	147.36	99.99%	99.31%	96.75%	48.3%
T1D	46,391,874	6,842,194,008	367,889	147.49	99.99%	99.31%	96.80%	49.91%
DL	45,324,868	6,683,378,913	334,016	147.46	100%	99.35%	96.95%	49.87%

Reads Count indicates the number of reads in the sample. Bases Count indicates the number of all bases. N Bases Count indicates the number of N bases. Average Length indicates the average sequence length. GC Ratio indicates the GC content. QC indicates the data quality control.

## Data Availability

All data generated or analyzed in this study are included in the main text.

## Supplementary Materials

The following supporting information can be downloaded at: https://www.mdpi.com/article/10.3390/biology14101343/s1, Figure S1: The original western blot images. Figure S2: The original western blot images.
