# Supplementary material for: Study on the Mechanism of Low-Intensity Pulsed Ultrasound in Ameliorating Glucose Metabolism Through Attenuation of Skeletal Muscle Atrophy in Mice with Type 1 Diabetes
Source: Biology (Basel). 2025 Oct 1;14(10):1343. doi: 10.3390/biology14101343 (PMC12561218; doi:10.3390/biology14101343)

Supplementary figures of western blot

Figure S1

Housekeeping proteins **Objective proteins**

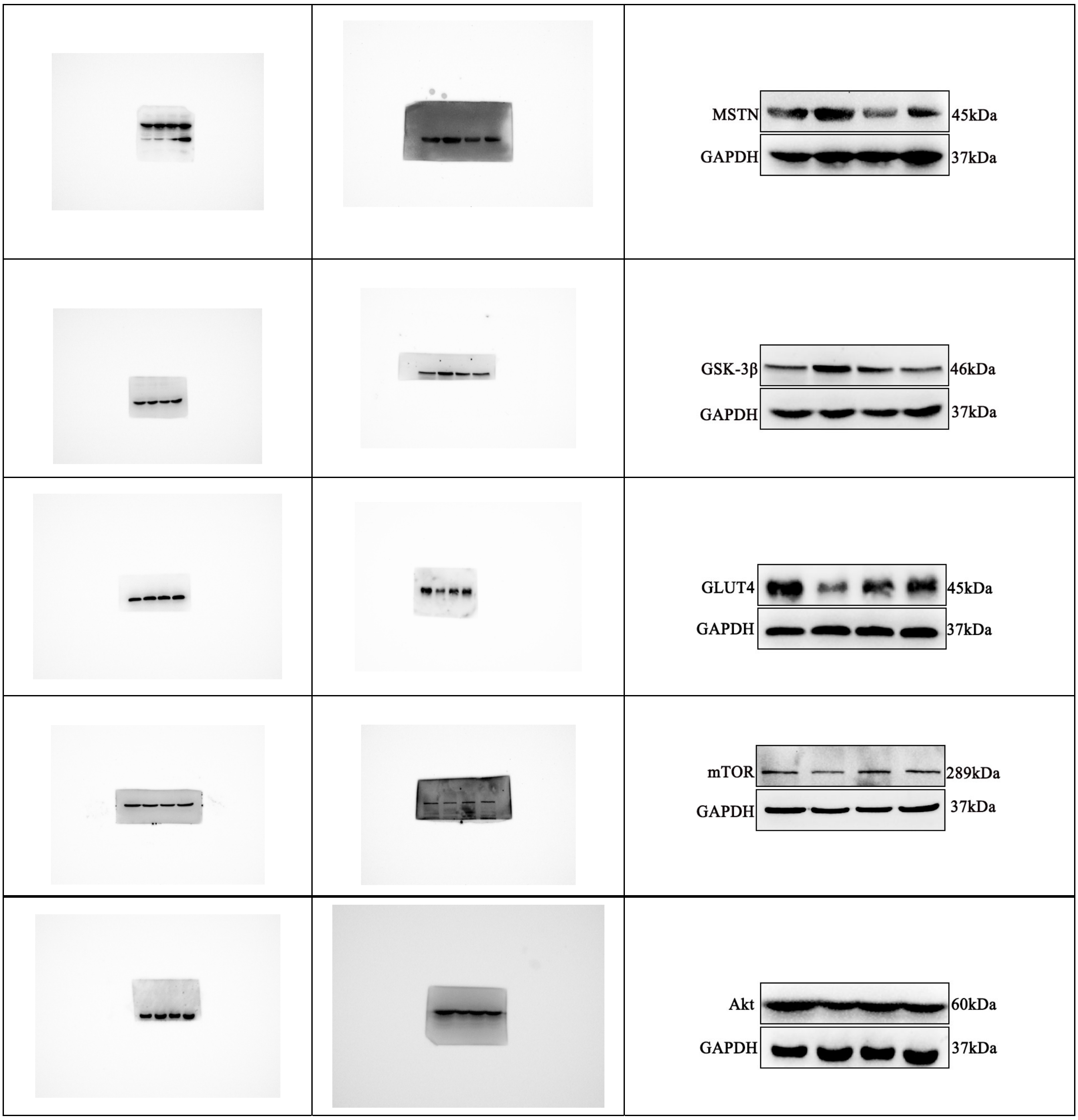

Figure S2

Housekeeping proteins **Objective proteins**

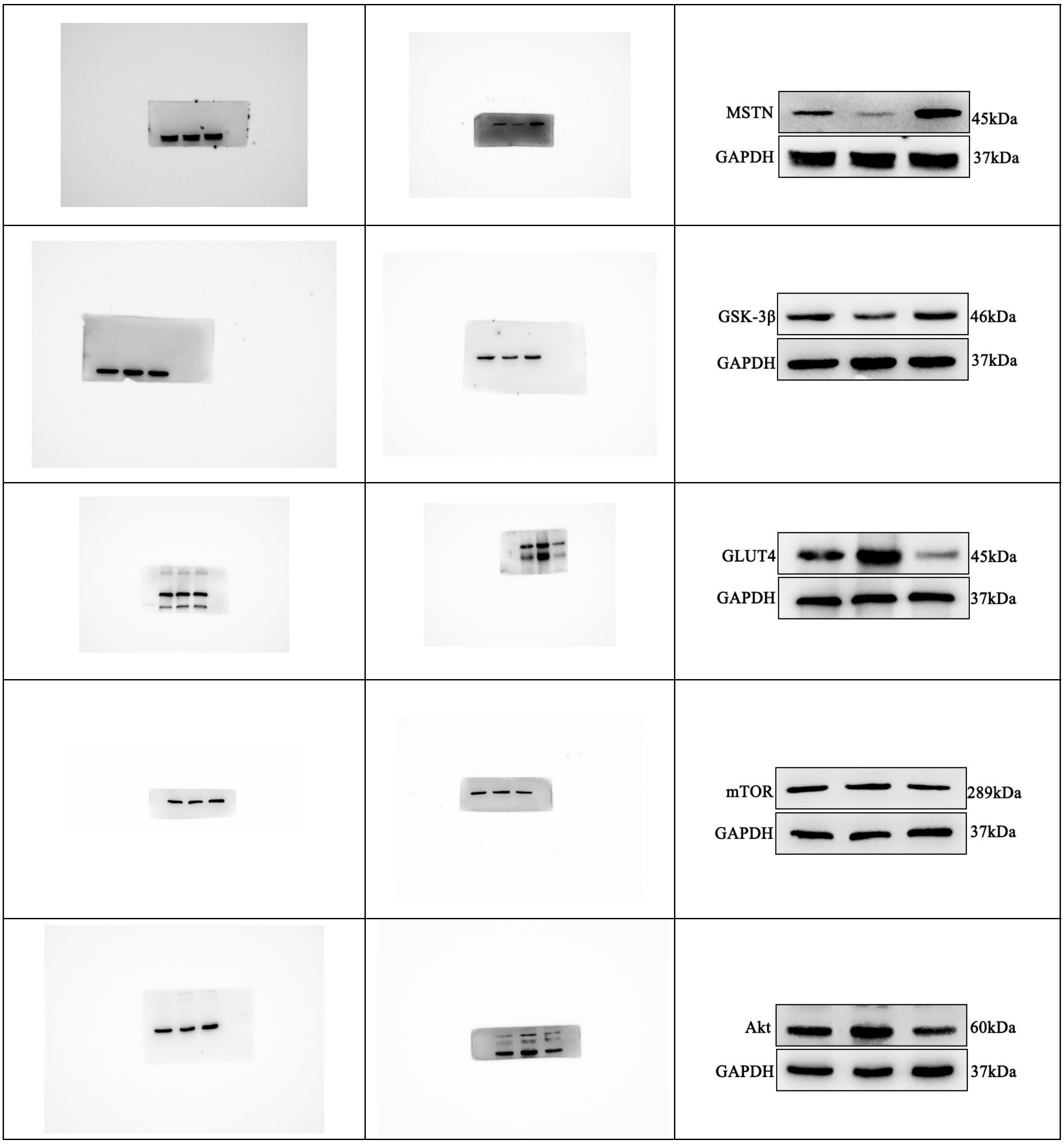

Supplement: Supplementary file 1 [file biology-14-01343-s001.zip › biology-3824073-supplementary.pdf]
